# When You Choose but Not Lose: Decreasing People’s Desire for Options on Technological Appliances

**DOI:** 10.3389/fpsyg.2022.749772

**Published:** 2022-02-25

**Authors:** Nieke Lemmen, Thijs Bouman, Linda Steg

**Affiliations:** Department of Psychology, Faculty of Behavioural and Social Sciences, University of Groningen, Groningen, Netherlands

**Keywords:** options, user frustration, choice-overload, loss aversion, technology design, Sustainable energy transition, sustainable energy system

## Abstract

The appliances people adopt, and the way they use them, can critically influence the sustainable energy transition. People are often attracted to appliances with many setting options that offer them more control. Yet, operating many setting options can have negative consequences for users (e.g., user frustration) and the management of sustainable energy systems (e.g., unpredictable consumption increasing complexity and uncertainty of systems), which may obstruct sustainability goals. We aim to study how to reduce the preference for many setting options without reducing the perceived attractiveness of the appliance. In line with our theorizing we found that people opt for appliances with fewer setting options when they are asked to reflect on which options they would like to have from a list of possible setting options, while being equally satisfied with the appliance. In addition, we show that this is especially the case when asking people to select which setting options they would like an appliance to have, as this will feel like they gain options, rather than asking them which options they are willing to give up as this feels like losing options that their appliance could have. Our findings offer relatively easy and cost-efficient ways to decrease people’s desire for many setting options on appliances, decreasing stress on the user and the energy system, while ensuring satisfaction with and acceptance of the appliance.

## Introduction

People play a central role in energy systems and, therefore, in achieving a sustainable energy transition. Households were responsible for 26.3% of the direct energy consumption in Europe in 2019 (Eurostat, 2019). People can contribute to a sustainable energy transition by accepting and adopting sustainable energy technologies, and using these in a sustainable way (Gram-Hanssen, 2011). Moreover, they can match their energy demand to the available (renewable) energy supply, to enhance the efficiency and sustainability of the energy system (Steg et al., 2021).

About 14% of household energy consumption is associated with the use of electrical appliances (Eurostat, 2019). Therefore, it is important to understand what motivates people to use appliances in a sustainable way. Notably, many appliances offer an increasing number of choice options, which could have implications for the sustainability of energy systems. For example, household appliances such as washing machines typically have a wide range of setting options, which affects energy use patterns. Users can choose to use shorter or lower temperature cycles, which consumes less energy or set the exact time they would like the load to be finished creating more flexibility in use. Even though more setting options give people more control over their appliances – something they typically find appealing (Leijten et al., 2014) – this also increases the complexity of and uncertainty for managing sustainable energy systems, as energy usage will now not only depend on how often and when an appliance is used, but also on which of the many setting options are selected.

In addition, while people evaluate appliances that offer more control as more acceptable and attractive (Leijten et al., 2014), too many choice options has been associated with a reduction in satisfaction (Iyengar and Lepper, 2000) as people feel overwhelmed by the number of choices. This implies that managing many options can be bothersome, frustrating, and could result in ineffective and inefficient use of the appliance, and put – often unnecessary – stress on the user (e.g., having to make many choices) and energy system (e.g., difficult to manage the energy system in a sustainable way because user behavior is less predictable). Indeed, several studies have found that programmable thermostats that provide more choice options to users are oftentimes not used (Peffer et al., 2011) or used in an inefficient way, as they have the radiators turned on for more hours (Guerra-Santin and Itard, 2010) and do not keep lower temperatures (Shipworth et al., 2010).

To achieve a sustainable energy system and improve user satisfaction, it thus seems important to examine whether and when people could be satisfied with having less choice options on their appliances. This is the key question we will study in this paper. Specifically, we aim to study whether people can be (equally) satisfied with appliances with fewer setting options. Notably, we test two strategies that make people reflect on which setting options they would like an appliance to have and examine whether and how this may reduce their apparent desire for having many setting options on an appliance, without reducing the attractiveness of the appliance.

First, we reason that people often rely on a heuristic that “more options is better” when making choices, while neglecting the potential negative consequences of having many options. Indeed, people often prefer to have many choice options (Iyengar and Lepper, 2000; Botti and Lyengar, 2004), but many options can also cause frustration, choice-overload (although this has not been found consistently across studies; Scheibehenne et al., 2010), and regret when people actually have to deal with many choice options (Schwartz, 2004; Heitmann et al., 2007; Sagi and Friedland, 2007). For example, people typically prefer large arrays of products to choose from in supermarkets, rather than a smaller selection. Yet, having many choices makes the decision-making process more complicated, and may lead to lower satisfaction with the chosen product (Iyengar and Lepper, 2000). Similarly, there is some initial evidence to suggest that people prefer to have many setting options when choosing an appliance, but get frustrated when using appliances with many setting options because it requires them to continuously make choices (Lemmen et al., 2017).

A key question is whether people can be satisfied with appliances with fewer setting options, which would be less frustrating to use. We propose that people will generally desire fewer options on their appliance when they actively reflect on the amount of setting options that they would like an appliance to have. We reason that doing so will make them consider the possible consequences and necessity of each possible setting option and will prevent that they merely rely on the heuristic that having more options is better. Consequently, they will be more likely to opt for fewer options on their appliance than ultimately possible. Hence, we hypothesize that asking people to reflect on the number of options they would like to have on an appliance from a list of possible setting options will make them opt for fewer options than ultimately possible (Hypothesis 1).

Second, we propose that *how* people are asked to select which options they like their appliance to have will affect the number of options they choose. Specifically, based on prospect theory, we reason that people’s preferences and choices are influenced by their reference point, as this affects the likelihood that they anticipate feelings of losses or gains (Kahneman and Tversky, 1979). People are more strongly motivated to avoid potential losses than they are to secure equal gains (Tversky and Kahneman, 1991, 1992). This suggests that when selecting options, people will likely select more options when they feel like they lose options that their appliance could have had, than when they feel they gain options. Therefore, we reason that people will select fewer options when they are asked to indicate which options they would like to have on their appliance than when they are asked to indicate which options they are willing to give up, as the former is likely to be experienced as gaining options while the latter is experiences as losing options. Accordingly, we hypothesize that people select fewer setting options when asked to select the options they want to have on their appliance (i.e., gain), compared to when they are asked to select the options they do not want on their appliance (i.e., loss) (Hypothesis 2).

We further propose that people will find their selected appliance equally attractive when they choose the number of options on the appliance themselves compared to when they are offered an appliance with many (more) options, because people are motivated to justify and rationalize their choices. Specifically, after making a decision, people tend to convince themselves that they made the right choice (Cialdini, 2001; Cushman, 2020). This implies that when people choose the number of options on their appliance themselves, they are motivated to justify their choice and hence be as satisfied with the resulting appliance as people who are offered an appliance with all possible options (Hypothesis 3).

## Method Study 1

### Participants

We conducted a paper-and-pencil questionnaire study among a sample of 132 students of the University of Groningen (59.8% female and 40.2% male), who were between 18 and 34 years old (*M* = 21.52, *SD* = 2.63). Students were approached at the break rooms and cafés of the University Library^1^.

### Procedure and Design

We used a between-subject design, in which we randomly assigned participants to one of two experimental conditions. All participants were asked to imagine they wanted to buy a washing machine and that they could choose which setting options this washing machine would have. They were provided with a list of 14 possible setting options (see Table 1) and were asked which setting options they would want to have on their washing machine. We systematically varied the framing of the question: participants were either asked to cross out the options that they did *not* want their washing machine to have (*n* = 66), or to circle the options they *would* want their washing machine to have (*n* = 66). We counted the total number of setting options that each participant chose. Moreover, respondents indicated how much they liked the washing machine that had the setting options they just chose, by asking them to indicate on a scale from 1 (*strongly disagree*) to 7 (*strongly agree*) to what extent they agreed with the statements: “I like this washing machine very much” and “I would like to have this washing machine.” Both items formed a reliable scale (ρ = 0.76) so we computed mean scores on these items, which we used in our analyses.

**TABLE 1:** Overview of the 14 setting options that participants could choose.

• The temperature of the water
• The duration of the program
• The scheduled end time of the program
• At what time you want your washing machine to start the washing program
• Whether you will wash white or colored laundry
• The spin speed (centrifuge)
• Whether you want the washing machine to use extra water or not
• Whether you want the washing machine to make a sound to signal it is finished
• Whether your laundry is delicate (e.g., wool) or not
• Whether you want your laundry to be pre-soaked or not
• Whether you want a pre-wash or not
• Whether you want wrinkles to be flattened out or not
• Whether you are able to stop the washing program during the cycle
• Whether you will wash a full load or half a load of laundry

Next, we aimed to explore whether choosing more or fewer options would affect participants’ evaluation of other characteristics of the washing machine. For this purpose, participants indicated on a scale from 1 (*not at all*) to 7 (*very much*) to what extent they thought the washing machine with the selected options: “is pleasant to use frequently,” “offers me a lot of control,” “can be set to my specific preferences,” “is frustrating to use frequently,” “has setting options I do not want to have,” “is annoying when used frequently,” “misses setting options I would like to have,” “is effective in removing stains,” and “is unpleasant to use frequently,” respectively. The negatively formulated items were reverse coded so that a higher score depicts a more positive evaluation of the washing machine. In addition, participants indicated whether the selected washing machine had “*far too few options*” (1) or “*far too many options*” (7), with “*just the right amount of options*” (3) as the midpoint of this scale. After filling out the questionnaire, participants were offered a flyer with a QR-code linking to a webpage that contained a debriefing. The study was approved by the Ethical Committee of Psychology of the University of Groningen.

### Results and Discussion

**FIGURE 1:**
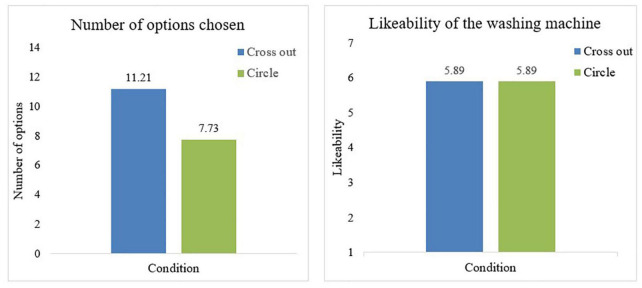
The mean number of options chosen and likeability of the chosen washing machine per condition.

In line with Hypothesis 1, we found that participants in both the cross-out condition (*M* = 11.21, *SD* = 2.17) and the circle condition (*M* = 7.73, *SD* = 2.41) choose significantly fewer options than ultimately possible [14; *t*(65) = −10.42, *p* < 0.001 and *t*(65) = −21.10, *p* < 0.001, respectively]. This finding suggests that asking people to think about which setting options they would like their appliance to have, by asking them to choose the amount of options they would like to have, can indeed make them opt for fewer options than possible. In addition, Figure 1 shows that participants who were asked to circle the options they would want their washing machine to have choose significantly fewer options than participants who were asked to cross out the options they did not want their washing machine to have [*t*(130) = −8.71, *p* < 0.001]. This finding supports Hypothesis 2 and shows that *how* you ask people to consider which setting options they want their appliance to have affects the amount of setting options they choose. Specifically, people seem to choose more options when they feel they lose options compared to when they feel they gain options.

Despite the difference in the number of setting options chosen across conditions, we found no differences in the extent to which participants in both conditions liked the appliance [*M* = 5.89 and *M* = 5.89, respectively; *t*(130) = 0.052, *p* = 0.959]. This finding offers initial support for Hypothesis 3 that there is no difference in likeability of appliances that have fewer options if people can freely choose which options the appliance would have. In addition, we found no significant differences in the other evaluations of the washing machine between the two conditions, with the exception of perceived necessity of setting options: participants in the cross out condition were significantly more likely to think that their washing machine had options that they did not necessarily need (*M* = 2.58) compared to participants in the circle condition [*M* = 2.03, *t*(130) = −2.27, *p* = 0.025]. These findings suggest that asking people to circle options they would like to have on their washing machine can lead people to opt for fewer options compared to asking them to cross of options they do not want, while they evaluate the washing machine equally positively.

Study 1 provides support for all three hypotheses. However, while participants in both conditions choose fewer setting options than ultimately possible, they were able to choose from a relatively large number of setting options. The question remains whether we will find the same results if we offer participants fewer options to begin with. Second, as we did not include a control condition, the question remains whether participants in both conditions liked the appliance equally well as people who were offered an appliance with all possible setting options. We conducted Study 2 to address these issues.

## Method Study 2

In Study 2 we test the same hypotheses as in Study 1, but we made a few modifications to the research design. First, we provided participants with only six possible setting options to choose from. To be sure all options were relevant to participants, we used the six setting options that were chosen most often in Study 1 (see Table 2). In addition, we added a control group in which participants imagined the washing machine would have all 6 setting options, so they were not asked to reflect on which setting options they would like the appliance to have.

**TABLE 2:** Overview of the six setting options that participants could choose.

• The temperature of the water
• The duration of the program
• The scheduled end time of the program
• Whether you will wash white or colored laundry
• The spin speed (centrifuge)
• If you want the washing machine to use extra water or not

### Participants

We conducted a paper-and-pencil questionnaire study among a sample of 160 students of the University of Groningen (64.4% female, 33.8% male and 3 participants who did not indicate their gender), who were between 18 and 58 years old (*M* = 22.91, *SD* = 5.65). Participants were approached to participate in our study in the break rooms and cafés of the University Library.

### Procedure and Measures

We followed the same procedure as in Study 1. Yet, this time, we added a control group (*n* = 35), next to the cross-out condition (*n* = 62), and the circle condition (*n* = 63). Participants in the control group were simply provided with the list of six setting options that the washing machine would have. To rule out that participants in the experimental conditions simply feel obligated to leave options out, we also we explored whether participants felt autonomous in choosing the options on their washing machine. We asked participants to indicate on a scale from 1 (*strongly disagree*) to 7 (*strongly agree*) to what extent they agreed with the following items: “I feel like I could choose which options this washing machine would have,” “I feel that I was free to decide for myself what options I wanted this washing machine to have” and “I had little control over the options the washing machine would have” (recoded). We computed mean scores on these items (*M* = 5.06, *SD* = 1.14; α = 0.66)^2^.

Next, as in Study 1, participants indicated to what extent they like the washing machine with the options presented to them (control condition) or selected by them (experimental conditions). Specifically, they indicated to what extent they agreed with the statements: “I like this washing machine very much” and “I would like to have this washing machine,” on a scale from 1 (*strongly disagree*) to 7 (*strongly agree*). These items formed a reliable scale (ρ = 0.73) so we computed mean scores on these items (*M* = 5.41, *SD* = 1.18). Moreover, we included four of the measures from Study 1 to explore whether participants across conditions differed in the evaluation of their chosen washing machines. Specifically, we asked them to indicate on a scale from 1 (*not at all*) to 7 (*very much*) to what extent they thought the washing machine offers them a lot of control, is frustrating to use frequently, has setting options they don’t want to have, and is unpleasant to use frequently, respectively.

### Results and Discussion

**FIGURE 2:**
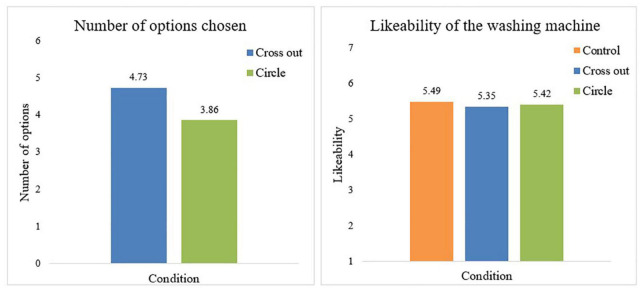
The mean number of options chosen and likeability of the washing machine per condition.

We again found support for Hypothesis 1: participants in both the cross out (*M* = 4.73, *SD* = 1.01) and circle condition (*M* = 3.86, *SD* = 1.08) chose significantly less options than the total amount of options (6) that were offered to participants in the control condition [*t*(95) = 7.44, *p* < 0.001 and *t* (96) = 11.76, *p* < 0.001, respectively]. Moreover, Figure 2 shows that, as in Study 1, participants who were asked to circle the options that they wanted their washing machine to have, chose significantly less options than participants who were asked to cross out options they did not want their washing machine to have [*t*(123) = −4.65, *p* < 0.001], supporting Hypothesis 2. Furthermore, Figure 2 shows that we did not find any differences in how much participants in each condition liked the washing machine [*F*(2,157) = 0.16, *p* = 0.850]. In addition, we found no significant differences in anticipated control over the washing machine [*F*(2,157) = 0.109, *p* = 0.896], anticipated frustration [*F*(2,157) = 1.60, *p* = 0.206] or unpleasantness of frequent use [*F*(2,157) = 0.33, *p* = 0.718] and perceived necessity of setting options [*F*(2,157) = 0.07, *p* = 0.935] between the three conditions. These findings provide further support for Hypothesis 3 suggesting that people who choose the number of options on their appliance themselves, will equally like this appliance as people who are offered an appliance with all possible options.

Next, we found that participants in all conditions felt autonomous in choosing the options on their washing machine as on average participants in the circle (*M* = 5.36, *SD* = 0.92), cross-out (*M* = 4.90, *SD* = 1.31) and control condition (*M* = 4.84, *SD* = 1.07) scored above the midpoint of the scale. Still, we did find a significant difference in feelings of autonomy between the three conditions [*F*(2,155) = 3.43, *p* = 0.035]. Planned contrast analysis showed that participants in the circle condition felt significantly more autonomous in choosing the options on the washing machine than both the control condition [*t*(63) = −2.39, *p* = 0.020] and the cross-out condition [*t* (110) = 2.23, *p* = 0.027]^3^. No significant differences were found in perceived autonomy of choosing the options between participants in the control condition and participants in the cross-out condition [*t* (83) = −0.24, *p* = 0.802]. These findings suggest that people who are asked to select options do not feel obliged to leave options out. In addition, it seems that people feel more autonomous in their decisions when they feel like they gain options compared to when they feel like they lose options and compared to not being offered a choice.

## General Discussion

Modern appliances typically offer people many setting options. While people generally prefer appliances that offer many options (Leijten et al., 2014), operating many setting options can have negative consequences for end users (e.g., user frustration; Lemmen et al., 2017) and the sustainable energy transition (e.g., inefficient use or unpredictable consumption increasing the complexity and uncertainty for managing sustainable energy systems). We aimed to examine whether people could be satisfied with having fewer setting options on appliances.

We first reasoned that people would select fewer options when they reflect on the options they would like an appliance to have, as this would prevent that they merely rely on the heuristic that having more options is better. As expected, in two studies, we found that participants who were asked to indicate which setting options they would like their appliance to have, chose significantly fewer options than possible. We found the same pattern of results when participants were asked to choose from a long list of setting options (14; Study 1) as when choosing from a list of fewer setting options (6; Study 2). These findings suggest that people select less options when they reflect on which options they would like an appliance to have, as doing so can prevent that people rely on the heuristic that more is better. Importantly, our results suggest that this is not because participants felt forced to select options, as people who were asked to select options did not feel less autonomous in choosing the options. In fact, we found in Study 2 that participants felt more autonomous in choosing the options when the task made them feel like they were gaining, compared to losing, options and compared to a control condition in which people were offered all options.

Second, we reasoned that how people are asked to select options on their appliances can affect the number of options they choose, as this may affect whether they feel like they gain vs. lose options. Specifically, we hypothesized that people would select fewer options when they have to indicate which option they would like the appliance to have compared to when they have to indicate which options they would give up (Hypothesis 2), as the former would be perceived as gaining options, while the latter would be perceived as losing options. As expected, in both studies we found that people selected fewer options when they had to circle the options they would like the appliance to have, which was likely experienced as gaining options, compared to when they had to cross out options they do not want, which is likely experienced as losing options. This finding is in line with prospect theory that proposes that preferences vary depending on people’s reference point, and that people are generally more strongly motivated to prevent potential losses than they are to secure equal gains (Kahneman and Tversky, 1979; Tversky and Kahneman, 1991). Yet, we extend previous research on prospect theory by showing that loss aversion may also impact the number of options people choose on appliances.

We further reasoned that selecting fewer options would not affect how satisfied people are with the appliance (Hypothesis 3), as people are motivated to rationalize their choices and convince themselves that they made the right choice (e.g., Cialdini, 2001). As expected, we found that participants who could choose which options their washing machine would have evaluated the washing machine equally positive as participants who were simply offered (and therefore could not choose) all possible setting options on their washing machine. This supports our reasoning that people are likely to rationalize their choices, and therefore are as satisfied with the selected appliance as people who were offered all options.

### Limitations and Directions for Future Research

Our studies have some limitations that should be kept in mind when interpreting the results. First, we tested our Hypotheses among a sample of university students, focusing on one specific appliance: a washing machine. Future research is needed to test whether similar results would be found for other appliances and among other samples. Notably, it is possible that people who differ in age, income or lifestyle may have different preferences for options on appliances (e.g., see Hauk et al., 2018). Yet, although we could expect that mean scores on the variables of interests may differ across appliances and samples (e.g., mean number of options chosen and mean level of satisfaction), this is not likely to affect the differences across conditions or relationships between variables (cf. Bhushan et al., 2019). Yet, future research should test the robustness or our results using different appliances and samples. Second, we tested our hypotheses using a hypothetical scenario in which participants were asked to imagine buying a washing machine. Future studies could test whether participants would make the same decisions when they are in a store buying an actual appliance and determine whether and under which conditions we would find the same results.

### Practical Implications

Our findings have important practical implications, as our results suggest how to encourage people to choose appliances with less setting options without compromising their level of satisfaction with such appliances. Specifically, our results suggest it is important to trigger people to reflect on which options they would want or like, and to make them select which options they like rather than select which options they would not like. Doing so may prevent people from selecting appliances with many options that can be frustrating when used. Moreover, this could result in higher engagement with the appliance over time and promote more efficient use. Furthermore, adopting appliances with fewer options would make it easier for grid operators to predict energy demand, which makes it easier to manage the energy grid.

Our results may have implications for the design of other energy technologies as well. For example, automated energy technologies are developed that can make choices on behalf of the user, which can increase the efficiency and sustainability of the energy system (Nguyen and Aiello, 2013). Yet, such technologies may be less acceptable because people are reluctant to hand over control (Leijten et al., 2014), which could reduce the efficiency and sustainability of the energy system. Our results show that offering many control options might not be necessary to secure acceptability of such technologies, but that it would be important to ask people to choose the options that they would like to control, as this is likely to make them select fewer control options without reducing their level of satisfaction with the technology. This would also offer possibilities for designing energy technologies with fewer options by leaving less relevant options out, which reduces costs. For example, stores could offer standardized basic appliances and offer consumers different types of features that they could add, thereby prompting them to reflect on which options they would like to have. In fact, this is already a common practice for car sales, in which people can opt for additional features at additional costs. Future studies are needed to test this further.

## Conclusion

Our studies show that there are relatively easy and cost-efficient ways to decrease end-users’ desire for many setting options on appliances, lowering the risk of potential user frustration, while ensuring satisfaction with and acceptance of the appliance. Specifically, asking people to reflect on the amount of setting options they would want their appliance to have can make them opt for fewer options than ultimately possible while being equally attracted to the appliance, particularly when asking people to select which setting options they would like an appliance to have, rather than asking them which options they are willing to give up.

## Data Availability Statement

The original contributions presented in the study are included in the article, further inquiries can be directed to the corresponding author.

## Ethics Statement

The studies involving human participants were reviewed and approved by Ethical Committee of Psychology of the University of Groningen. The participants provided their written informed consent to participate in this study.

## Author Contributions

NL and LS originated the research ideas and designed the study. NL collected the data, performed the data analysis, and drafted the manuscript. TB and LS engaged in several rounds of critical revisions of the manuscript. All authors approved of the final version of the manuscript to be published.

## Conflict of Interest

The authors declare that the research was conducted in the absence of any commercial or financial relationships that could be construed as a potential conflict of interest.

## Publisher’s Note

All claims expressed in this article are solely those of the authors and do not necessarily represent those of their affiliated organizations, or those of the publisher, the editors and the reviewers. Any product that may be evaluated in this article, or claim that may be made by its manufacturer, is not guaranteed or endorsed by the publisher.

## Footnotes

1 Almost all students who were approached, agreed to participate in the study, with only a few exceptions of students who turned out there be in a meeting or had to leave soon for a meeting or phone call.

2 We conducted the same analysis for each individual item and found the same pattern of results.

3 As Levene’s test for equality of variances was significant [*F*(121) = 8.12, *p* = 0.005], we reported the statistics for this analysis based on the assumption that the variance of feelings of autonomy was not equal between conditions.
